# Editorial: Advancing the development and implementation of regional, national tuberculosis control programs in livestock in Africa, Asia, and Latin America

**DOI:** 10.3389/fvets.2023.1192091

**Published:** 2023-04-18

**Authors:** Douwe Bakker, Joram Josephat Buza, Julio Álvarez, Vivek Kapur

**Affiliations:** ^1^Departamento de Sanidad Animal, Facultad de Veterinaria, Universidad Complutense, Madrid, Spain; ^2^School of Life Sciences and Bioengineering, The Pennsylvania State University (PSU), University Park, State College, PA, United States; ^3^Huck Institutes of the Life Sciences, Nelson Mandela African Institution of Science and Technology, Arusha, Tanzania; ^4^VISAVET Health Surveillance Centre (UCM), Madrid, Spain; ^5^Department of Veterinary Population Medicine, College of Veterinary Medicine, University of Minnesota, Saint Paul, MN, United States

**Keywords:** tuberculosis, Low- and Middle-Income Countries, diagnosis, surveillance, control, eradication, vaccination, cost benefit

## Introduction

Tuberculosis in livestock caused by members of the *Mycobacterium tuberculosis* (MTb) complex is a notifiable zoonotic animal disease (1), which has been eradicated or held to very low prevalence levels in many high-income economies. Successful campaigns were all build on a very strict test-and-slaughter strategy using the tuberculin PPD skin tests as diagnostic tool. However, tuberculosis in livestock remains endemic in most Low- and Middle-Income Countries (LMICs). This not only represents a threat to public health in those countries but also places a significant burden on their economies due to a negative impact on livestock productivity and the resources invested in healthcare, prevention, surveillance, and, when present, control and/or eradication programs. Moreover, tuberculosis in livestock affects a wide variety of species as well as breeds, raised in a wide variety of farming systems, in a broad range of different climates, thus ruling out a “one size fits all” approach for disease control. Since “traditional” test and cull programs are costly, very demanding on the livestock holder and may be ruled out as option for religious reasons, such programs must be tailored to ensure they are fit for purpose considering the respective socio-economic context in which they have to be implemented in each country.

This Research Topic includes a variety of articles addressing some of the aspects of the design, description, evaluation and monitoring economic benefits/costs of surveillance and/or eradication programs targeting tuberculosis in livestock in LMICs.

## Mammalian tuberculosis

In 2022 the World Organization for Animal Health (WOAH) replaced the chapter for Bovine Tuberculosis in their Terrestrial Animal Health Manual with a new chapter on Mammalian Tuberculosis, thereby recognizing the fact the original definition of bovine tuberculosis, which was restricted to tuberculosis in bovines caused by *Mycobacterium bovis* (Mb), was no longer fit for purpose. Tuberculosis in bovines was shown to be also caused by other recently characterized new members of the MTb complex whereas at the same time Mb was shown to cause tuberculosis in wide variety of other mammalian hosts.

Two articles in this Research Topic further illustrate this issue (Ambaw et al.; Barandiaran et al.). Barandiaran et al. investigates the epidemiology of tuberculosis in pigs in Argentina caused by a molecular diverse population of Mb strains, which share 60% of their genotypes with Mb strains isolated from local cattle and wild boar, suggesting transmission at the interface between pigs, cattle and wild boar. This is a further indication that the control of Tb in other susceptible mammalian hosts should not be ignored while attempting to control and eradicate Tb from bovines. In the second article by Ambaw et al. evidence is provided for a difference in disease susceptibility between three cattle breeds in Ethiopia. A significant difference in skin test reactivity and pathology severity between infected animals belonging to the respective breeds housed on the same farm was observed. Moreover, in addition to the 14 Mb isolates recovered from the lesions, two *M. tuberculosis* (M. tb) isolates were recovered from lesions as well. Thereby it is illustrating the fact that other members of the MTb-complex can cause tuberculosis in cattle with an identical pathology.

## Diagnostics and epidemiology

The diagnosis of tuberculosis in livestock as well as the detection and the characterization of its causative agents are described in detail in the aforementioned chapter of the WOAH Terrestrial Animal Health Manual (1).

The prescribed *ante mortem* tests used are the tuberculin intradermal tests, respectively the comparative cervical test (CCT), the single cervical test (SCT) and the caudal fold test (CFT), and the *in vitro* interferon gamma release assay (IGRA). Two of the articles in this Research Topic [Kelly et al. (a); Kelly et al. (b)] evaluate the use of both tests and the application of one of them, the IGRA, in sub-Saharan Cameroon. In Kelly et al. (a) cattle were sampled in two different regions and the results of the IGRA and the CCT were compared: in one of the regions the agreement between both tests was fair, whereas in the other region the agreement was poor-moderate. The main source of test disagreement was animals testing positive by IGRA and negative by CCT. The authors concluded that an individual or combined use of these tests could lead to a large variation in TB estimates in different settings and/or trials. The same authors subsequently used the IGRA in Kelly et al. (b) to describe the epidemiology of Tb in dairy herds in Cameroon and the potential public health risk from those dairy herds.

In a longitudinal study by Tschopp, Gemechu et al. the productivity of intensive dairy herds in Central Ethiopia was assessed and found to be sub-optimal. Subsequently, Tschopp, Conlan et al. used the CCT as a proxy of disease status to analyse the effect of tuberculosis on the productivity and movement of dairy cattle in and between those herds. Test negative bulls were shown to be heavier than reactor bulls indicating a production loss due to Tb infection. Interestingly, it was also noted that test-positive animals were eliminated faster from the herd than test-negative animals. Not by slaughter, or by being separated from the test-negative animals but often by selling those test-positive animals directly to other farms, sometimes further away to other regions, posing a significant risk for further spread of Tb. This highlights the need for stricter guidelines on the handling of Tb test positive animals and the urgent need for an animal identification and tracing system.

## Traditional control and alternative approaches

As mentioned before, the traditional way to control and eradicate tuberculosis from a livestock holding is the test- and-cull approach using the tuberculin skin test (1). To assess the feasibility of such an approach in Ethiopia, a pilot study was performed by Lakew et al. Over a period of 6 years all cattle in an infected farm were tested by CCT and all test-positive animals were culled. In the fifth and final round of testing the number of CCT positives had dropped to almost zero. However, analyses of the skin test data applying the single cervical test (SCT) cut-off's, indicated that there was still a considerable level of residual Tb infection present in this herd, indicating that a prolonged control by test-and-cull would be need. Moreover, the high costs of culling and replacing considerable numbers of test positive animals confirmed that the test-and-cull strategy may be an impractical approach for a nationwide implementation in Ethiopia as well in most other LMICs.

In two articles, potential alternatives for the test-and-cull approach are described. Mazorra-Carrillo et al. compared serum proteins from Tb infected and non-infected cows and identified three proteins involved in inflammatory/immunomodulatory responses to infections being expressed at a higher level in non-infected or potentially resistant cows. These proteins might be useful as novel biomarkers for the breeding of cattle resistant to TB. Finally, Sirak et al. looked at the difference in the expression of immunological markers and gross pathology between *M. bovis BCG* vaccinated and non-vaccinated cross-breed calves in Ethiopia. Findings indicated stronger responses of a set of immunological cells and markers at local granulomas as well as a reduction of the severity of the gross pathology at the primary site of infection in vaccinated animals, thus indicating that (repeated) BCG vaccination could play a role in the control of tuberculosis by a gradual reduction of Tb infection in vaccinated herds.

## Author contributions

All authors listed have made a substantial, direct, and intellectual contribution to the work and approved it for publication.
